# Neural Substrate of Body Size: Illusory Feeling of Shrinking of the Waist

**DOI:** 10.1371/journal.pbio.0030412

**Published:** 2005-11-29

**Authors:** H. Henrik Ehrsson, Tomonori Kito, Norihiro Sadato, Richard E Passingham, Eiichi Naito

**Affiliations:** **1**Wellcome Department of Imaging Neuroscience, Institute of Neurology, London, United Kingdom,; **2**Graduate School of Human and Environmental studies, Kyoto University, Sakyo-Ku, Kyoto, Japan,; **3**Department of Cerebral Research, National Institute for Physiological Sciences, Okazaki Aichi, Japan,; **4**Department of Experimental Psychology, University of Oxford, Oxford, United Kingdom,; **5**ATR Computational Neuroscience Labs, Hikaridai, Seika-Cho, Soraku-Gun, Kyoto, Japan; Brandeis UniversityUnited States of America

## Abstract

The perception of the size and shape of one's body (body image) is a fundamental aspect of how we experience ourselves. We studied the neural correlates underlying perceived changes in the relative size of body parts by using a perceptual illusion in which participants felt that their waist was shrinking. We scanned the brains of the participants using functional magnetic resonance imaging. We found that activity in the cortices lining the left postcentral sulcus and the anterior part of the intraparietal sulcus reflected the illusion of waist shrinking, and that this activity was correlated with the reported degree of shrinking. These results suggest that the perceived changes in the size and shape of body parts are mediated by hierarchically higher-order somatosensory areas in the parietal cortex. Based on this finding we suggest that relative size of body parts is computed by the integration of more elementary somatic signals from different body segments.

## Abstract 2

Functional magnetic resonance imaging (fMRI) during a perceptual illusion suggests that activity in the parietal cortex is important for the integration of body image information.

## Introduction

The term “body image” commonly refers to the perception of the spatial dimensions of the body, its size, shape, and relative configuration of its parts [1,2]. The perception of the size and shape of body parts is archetypical and an important aspect of the body image. Everyday examples in which information about body size is used include when feeling ourselves as thin or large, or when walking through a narrow doorway. Unlike more elementary bodily senses such as limb movement, touch, and pain, there are no specialized receptors in the body that provide information to the brain about the size and shape of body segments. Furthermore, the somatotopically organized maps of the body surface in the somatosensory cortex that receive the sensory inputs from the peripheral receptors do not contain any explicit information about the relative size of body parts. Thus, an important question in sensory neuroscience is how the central nervous system computes the relative size and shape of the body and its parts.

Psychophysical studies suggest that the perceived relative size of body parts depends on the integration and comparison of somatic signals from different body segments [3–5] and of visual information from the body [6]**.** The size of body parts is probably represented in a relative sense, that is, relative to the size of other body parts and objects in the external environment. These notions are supported by the fact that people can experience illusions that the size and shape of a body part is changing when the central nervous system receives conflicting sensory signals from different body parts [3–5,7]. Likewise, in the absence of afferent sensory inputs, for example during anaesthesia of a limb or after amputation, subjects sometimes perceive changes in the size and shape of body parts [8,9]. Under certain pathological conditions affecting the parietal cortex, such as stroke [10], epilepsy with parietal focus [11–13], or somesthetic auras during migraine [14–16], people can experience changes in the size and shape of one or several limbs and body parts. Although these studies suggest that the brain processes information about body size and shape, and indicate that the parietal lobes are involved, the underlying neuronal substrate and mechanisms remain uncertain.

**Figure 1:**
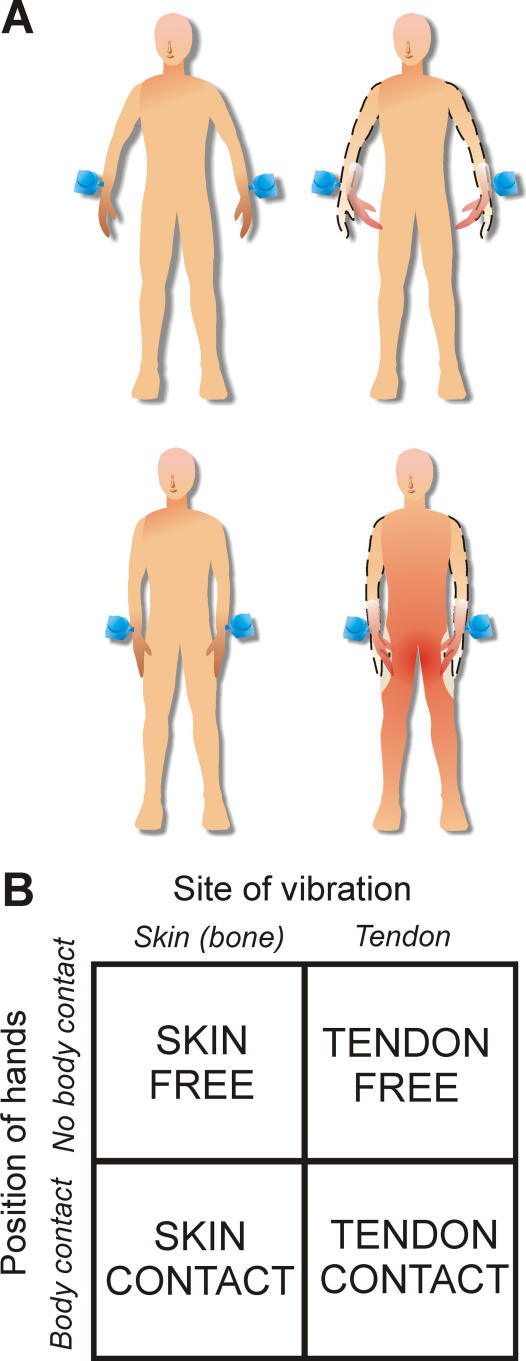
The Position of the Two Hands Relative to the Body and the Factorial Design of the Experiment When the palms of the hands were in contact with the body, the vibration of the two wrists elicited the illusion that the wrists were passively flexing and the waist and hips were shrinking (A, lower right). When the hands were not in contact with the body, the vibration of the wrists only elicited the illusion that the hands were flexing (A, top right). In two additional conditions, we vibrated the skin over the styloid bone beside the tendon, which does not elicit any illusions (A, top left and lower left). The neural effect of the shrinking-body illusion can be modelled as the interaction term between hand position and site of vibration in a 2 × 2 factorial design (see [B], [tendon contact– skin contact] – [tendon free – skin free]).

Here we used functional magnetic resonance imaging (fMRI) to investigate the neural correlates of perceptual changes of the size and shape of a body part. To experimentally manipulate the body image, we took advantage of a perceptual illusion—the “Pinocchio illusion” [4]—during which subjects feel that a body part changes its size and shape. This illusion has been demonstrated to work for both the length of the nose and for the width, height, and shape of various other body parts [4]. These illusions make use of the fact that vibration of the skin over the tendon of a joint extensor muscle elicits a vivid kinaesthetic illusion that the joint is passively flexing [17–21]. It is now well established that the illusory movements are caused by the excitation of muscle spindles in the vibrated muscle [18,22,23]. The afferent signals from this stimulation reach the primary somatosensory cortex [24–26] and primary motor cortex [19–21,26,27]. What Lackner [4] demonstrated was that if the hand is in direct contact with another body part, e.g., the nose or the waist, the subjects will not only feel that the vibrated wrist is bending but also experience the other body part being stretched or shrinking. In these situations the distortion of the body image is determined by the pattern of sensory stimulation according to a strict perceptual logic, so that the changes in shape and size of a body part appear to be caused by the illusory movement of the hand [4] (Protocol S1; Figures S1 and S2). For example, if the hand is grasping the nose and the biceps tendon is vibrated, one experiences the illusion that the hand is moving away from the face and the nose is becoming elongated. In contrast, when the triceps tendon is vibrated one feels that the hand is moving towards the face and that the nose is becoming shorter. We made use of the “waist-shrinking illusion.” The subjects put their hands so that the palms are in direct contact with the lateral sides of waist and the hips (see Figure 1). Then, when the tendons of the wrist extensor muscles are vibrated, the participants not only feel that the hands are bending inwards, but they also have the experience that the waist and the hips are shrinking.

We hypothesised that activity in higher-order somatosensory areas in the parietal cortex would reflect the perceived changes in waist size. These areas receive somatic information from different body parts [7,28–32] and thus, theoretically, have the capacity to integrate this information to compute the relative size of different body parts.

To reveal the activity associated with the feeling of waist shrinking, we used a 2 × 2 factorial design where the illusion was modelled as the interaction between hand position (attached to body [contact]/not attached [free]) and site of vibration (wrist tendon [tendon]/skin beside the tendon [skin]); see Figure 1 and Materials and Methods]. Before the brain scan commenced, we tested all subjects to make sure that they experienced a strong and reliable shrinking-waist illusion (see Results and Materials and Methods). Also, during these test sessions we quantified the illusion and we later used this measure to relate the strength of the illusion to the brain activity.

## Results

### Psychophysics

Pilot experiments on 24 participants had shown that the shrinking-waist illusion starts quickly, persists throughout a 30-s period of stimulation, and can be repeatedly elicited for as many times as required for the fMRI experiment. These initial observations were confirmed by the psychophysical experiments before the brain scans.

All 17 subjects that participated in the brain scan reported that they felt as if their hands were flexing passively and their waist shrinking during the tendon contact condition when asked to describe their experiences without leading questions. When asked to select a picture out of six pictures showing different conceivable body-image distortions, all subjects selected the picture showing that the waist was shrinking and the hands were bending towards the body (see Materials and Methods). It is important to note that participants did not report the sensation that the hands were moving into the body.

The participants rated the vividness of the shrinking body illusion as 6.8 ± 1.7 (mean ± standard deviation [SD]: rating from 0–9) and continuance as 8.1 ± 1.3 (mean ± SD) in tendon contact. They also reported that the illusion started after 3.3 ± 2.0 s (mean ± SD) of vibration. Thus, the illusion of waist shrinkage was vivid and reliable, and it started quickly. Furthermore, the quantification of the illusion showed that the subjects experienced that their wrists flexed by 13.6° ± 7.5° (mean ± SD for right and left wrist). This corresponded to a reduction in waist width by 9.4 ± 3.5 cm (mean ± SD), which is a 28% reduction in width.

We also quantified the illusion of wrist movement in the condition in which the hands did not touch the body (tendon free). Consistent with previous studies [21], they felt that their wrists were flexing by 22.9° ± 15.4° (mean ± SD) in this condition. The fact that the illusory wrist movements were greater in this control condition than in the shrinking-waist condition was expected because hand contact with body parts tends to reduce the wrist illusions [21].

Finally, the electromyograms (EMGs) showed no muscular activity in 12 of the 17 subjects. Five subjects showed some weak (<150 μV; in the order of 1%–3% of maximal voluntary contraction) and brief (<5 s) EMG responses. These activities were recorded both in the extensor carpi radialis (ECR) and flexor carpi radialis (FCR), and did not seem to have any relationship with the illusions. Importantly, the muscle activity was not significantly different in tendon contact and tendon free when we performed a quantitative integrated EMG analysis (*p* > 0.05 paired *t*-test; see Figure S3). Thus, the weak muscular activity that occurred in some subjects was matched in the comparisons between the tendon conditions and could not therefore have influenced our imaging results.

### Brain Imaging

**Figure 2:**
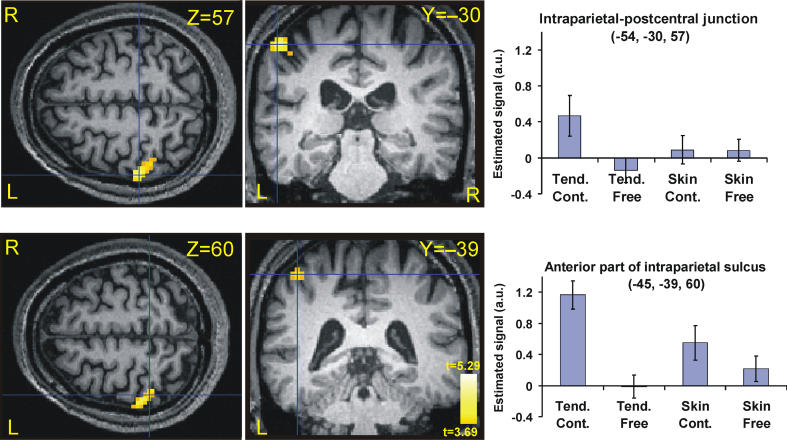
Activity that Reflects the Illusion that the Waist Was Shrinking (Interaction Effect: *p* < 0.001 Uncorrected) Top row: Activation of the cortices lining postcentral sulcus near its junction with the intraparietal sulcus. Lower row: Activation of cortex lining the intraparietal sulcus. The activations (colour) are superimposed on a normalized high-resolution T1-weighted image of a representative participant (black and white). The coordinates for the displayed slices are shown, and the crossing of the blue lines indicates the location of the activation peaks. R and L denote the right and left hemispheres, respectively. The plots to the right show the contrast estimates with the standard bars corresponding to the standard error (SE).

First, we analysed the activity that reflected the shrinking-body illusion that could not be attributed to the effects of vibrating the wrist tendon or the position of the arms. This activity is given by the interaction term in the factorial design ([tendon contact – skin contact] – [tendon free – skin free]) (see Methods and Methods; Figure 1). We found one cluster of active voxels in the whole brain that was located in the left parietal lobe (size: 200 mm^3^; *p* < 0.05 corrected; see Figure 2). The cluster was located in the anterior part of the intraparietal sulcus and extended rostrally to the postcentral sulcus. This cluster contained two distinct peaks of activation (Figure 2). The anterior peak was located in the postcentral sulcus (*x* = −54, *y* = −30, *z* = 57 [*x, y,* and *z* coordinates in the standard space of the Montreal Neurological Institute]; *t* = 4.76; *p* < 0.001 uncorrected) near the junction of this sulcus and the intraparietal sulcus. The posterior peak was located at the border between the superior parietal convexity and the anterior part of intraparietal sulcus (*x* = −45, *y* = −39, *z* = 60; *t* = 3.86; *p* < 0.001 uncorrected). In the right hemisphere, there was a statistical trend for activation in the corresponding parietal sites (intraparietal sulcus: *x* = 42, −30, 60; *t* = 2.91; *p* = 0.005 uncorrected; superior parietal gyrus: *x* = 48, *y* = −33, *z* = 72; *t* = 3.15; *p* = 0.003; not shown in the Figures). The activity in the two left parietal foci was not significantly greater than the activity in the right corresponding areas (*p* > 0.19 uncorrected). Hence, the parietal cortex appears to be bilaterally engaged, albeit with only a statistical trend for activation on the right side.

When we analysed the interaction term that reflected the shrinking-waist illusion, no activation, not even at a very low significance level (*p* < 0.05 uncorrected), was detected in the primary somatosensory cortex (areas 3a, 3b, and 1), the parietal operculum (SII), or the primary motor cortex (areas 4a and 4p). This observation supports our notion that the experimental design successfully matched the effects related to skin vibration, tendon vibration, and arm position. When these effects are not carefully matched, we know from our earlier studies that these areas are activated [21].

**Figure 3:**
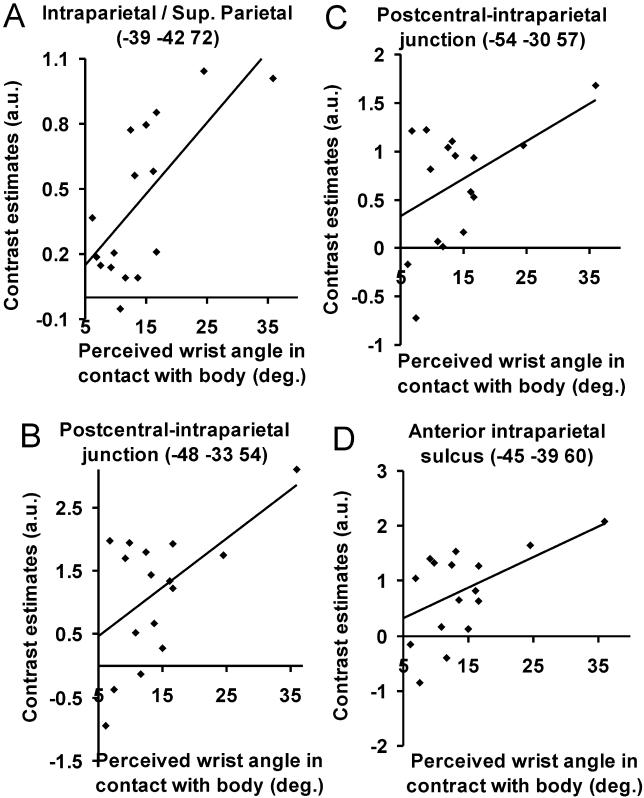
Linear Relationship between Parietal Activity and the Strength of the Shrinking-Waist Illusion Each dot represents the values for one individual subject. The data is fitted with a least-squares regression line. In (A) and (B) we plot the activity from the peaks in the intraparietal region that showed the most significant relation between illusion strength and neuronal activity ([A]: *x* = −39, *y* = −42, *z* = 72, *t* = 3.81; *p* < 0.001 uncorrected, *R* ^2^ = 0.4916, Pearson's *R* = 0.70; [B]: *x* = −48, *y* = −33, *z* = 54; *t* = 2.69; *p* < 0.009 uncorrected, *R* ^2^ = 0.32, Pearson's *R* = 0.57). These peaks were identified by using SPM2 to search for parietal voxels using a second-level linear regression model. (C) and (D) show the relationship between illusion and activity at exactly those peak voxels detected in the interaction analysis (see Figure 2). (C) shows the cortex at the junction between the postcentral sulcus and the intraparietal sulcus (*p* < 0.027, *y* = 0.0385x + 0.1424, *R* ^2^ = 0.23, Pearson's *R* = 0.48), and (D) illustrates the anterior part of the intraparietal cortex (*p* < 0.016, *y* = 0.0552x + 0.0488, *R* ^2^ = 0.27, Pearson's *R* = 0.52). In all plots the *y*-axis indicates the BOLD response (contrast estimates for interaction effect) in the parietal cortex, and the *x*-axis indicates the illusory displacement of the wrists when in contact with the body (which corresponds to the degree of waist shrinking). These regressions are not driven by outliers because all four remained significant (*p* < 0.05) when we used a least-square fitting procedure that minimizes the effects of outliers (Robustfit in MATLAB; see Results for details).

Next, we investigated whether there was a relationship between the activity in the parietal cortex and the strength of the body-image illusion. Because we had quantified the strength of the illusion for each subject in the test sessions prior to the scans, we could examine how the blood oxygenation level-dependent (BOLD) signal in the parietal cortex related to these illusion ratings. First, we used a linear regression model to search for voxels in the left intraparietal cortex in which the activity was related to the degree of body shrinkage across subjects (Figure 3). We found a peak of activation in the most anterior part of the intraparietal sulcus (Figure 3B; *x* = −48, *y* = −33, *z* = 54; *t* = 2.69; *p* < 0.009 uncorrected, *R* ^2^ = 0.32, Pearson's *R* = 0.57). This peak was located within the cluster of active voxels detected in the factorial design above, and therefore it probably corresponds to the same area. Also just adjacent, we found a peak in the border zone between the anterior part of the intraparietal sulcus and the left superior parietal gyrus (Figure 3A; *x* = −39, *y* = −42, *z* = 72, *t* = 3.81; *p* < 0.001 uncorrected, *R* ^2^ = 0.4916, Pearson's *R* = 0.70). Second, we examined exactly those peak voxels that were detected in the interaction analysis. As shown in Figure 3C and 3D, there was a linear relationship between the degree of illusory waist shrinking and the BOLD activity at these sites (*p* < 0.05 uncorrected). Taken together, these findings demonstrate that the subjects who reported the strongest shrinking-waist illusion also showed the strongest BOLD signal in the left postcentral sulcus and in the anterior part of left the intraparietal cortex.

To ensure that these results were not dominated by outliers, we used a linear regression analysis with a modified least-squares algorithm that is much less sensitive to outliers (Robustfit in MATLAB 6.5). This algorithm gives lower weight to points that do not fit well. Using this approach we found significant relationships between the BOLD signal and the illusion in all four regions presented in Figure 3: Figure 3A (*x* = −39, *y* = −42, *z* = 72): *p* < 0.001 (one-tailed); *t* = 3.54, *df* = 15; Figure 3B (*x* = −48, *y* = −33, *z* = 54): *p* < 0.025 (one-tailed); *t* = 2.45, *df* = 15; Figure 3C (*x* = −54, *y* = −30, *z* = 57): *p* < 0.05 (one-tailed); *t* = 1.864, *df* = 15; Figure 3D (*x* = −45, *y* = −39, *z* = 60): *p* < 0.05 (one-tailed); *t* = 1.94, *df* = 15. This means that the regression was not driven by outliers.

Finally, no activity was observed in the intraparietal or postcentral sulci when the subjects felt illusory wrist flexion when their hands were not touching the body (i.e., no waist-shrinking illusion; tendon free − skin free; *p* < 0.001 uncorrected) As in the previous experiments of Naito et al. [19,21,33], the illusory hand movements in the present study activated the bilateral primary motor cortices, dorsal premotor cortices, supplementary motor areas, right inferior parietal cortex, right inferior frontal cortex, and bilateral cerebellum (*p* < 0.001 uncorrected; some of these regions are shown in Figure S4).

## Discussion

Taken together, our results show that neural activity in the parietal cortex reflected the illusory sensation that the size and shape of the waist were changing. This illusion is elicited when the hands are in contact with the waist and the tendons of both hands are vibrated. Thus in this situation the brain receives conflicting sensory information from the vibrated wrists and the contact surfaces between the hands and waist. The input from the vibrated wrist muscles signals to the brain that the hands are flexing, whereas the tactile signals from the palms remained stable, signalling that the hands were in contact with the waist and hips. This conflict is resolved by recalibrating the size and shape of the waist and hips, so that it feels as if the waist/hip region is shrinking as the hands are bending inwards. We found activity in the cortices lining the left postcentral and left intraparietal sulci reflecting the shrinking-waist illusion. Furthermore, there was a linear relationship between the level of activity in these parietal areas and strength of the illusion across subjects. In other words, the subjects that reported the strongest illusion in the psychophysical test session also displayed the strongest parietal activity. The activity in the cortices lining the postcentral and anterior intraparietal sulci probably reflects the neuronal computations associated with the recalibration of the size and shape of the waist. Thus, these parietal areas are likely to be important for the construction of the body image.

The parietal activity can not be explained in terms of the sensory stimulation or the different postures of the arms and hands. The effects of vibrating the skin and the wrist muscles and changing the arm postures were matched in the factorial design ([tendon contact – skin contact] – [tendon free – skin free]), as were the effects related to the kinaesthetic illusions of wrist movement. Furthermore, illusory wrist movements do not activate the parietal areas in question (*p* > 0.05 uncorrected), but rather activate other areas such as the primary motor cortex [19–21] (see also Figure S4).

The activity associated with the waist-size illusion lies in the postcentral sulcus at its junction with the intraparietal sulcus and in the most anterior part of the intraparietal sulcus. This activity is located in the border region between somatosensory area 2 and the intraparietal sulcus (http://www.bic.mni.mcgill.ca/cytoarchitectonics/), but probably anterior to AIP (the anterior intraparietal area) [34]. The sulcal cortex anterior to AIP responds to somatic stimulation [34]. In the monkey brain, somatosensory area 2, and area 5 which lies posterior to area 2, are considered to be higher-order somatosensory areas [32,35,36]. Cells in these areas are active when different limbs and other parts of the body are touched or moved, i.e., they have complex receptive fields that include several body parts [28,32,35,36]. For example, some cells discharge when the hand, arm, or torso are touched [28], and many cells have bilateral receptive fields [37]. Such cells are not found in the primary somatosensory areas 3a, 3b, or 1. Thus, the neuronal populations in the cortices lining the postcentral sulcus and anterior part of the intraparietal cortex have the capacity to integrate tactile and proprioceptive information from different body parts. Because the shrinking-body illusion depends on the integration and interpretation of somatosensory inputs from different body parts, the postcentral and intraparietal activity could reflect this integration and recalibration process. This result supports the general hypothesis of hierarchical processing in the somatosensory system [32] and extends this principle to the representation of the body image. Afferent inputs from skin, joints, and muscles primarily reach the primary somatosensory cortex [24–26,38,39] and the primary motor cortex [19–21,27,40–42]. From these somatotopically organized primary representations (areas 4, 3a, 3b, and 1), somatic signals from different body parts converge onto higher-order somatosensory regions where the neuronal computations critical for the recalibration of body-part size may be performed

In our previous studies [21,43], we found activity in the right inferior parietal cortex (supramarginal cortex) when the subjects experienced illusory movements of the right hand, left hand, or both hands. In the present study we also observed activity in the right supramarginal cortex both during the illusion of both hands bending (tendon free– rest; *p* < 0.001 uncorrected) and during the illusion that the hands were bending and the waist was shrinking (tendon contact– rest; *p* < 0.001 uncorrected). Thus, the inferior parietal activation was eliminated when we examined the interaction term to look for activity specifically associated with the shrinking-waist illusion. This means that the right inferior posterior parietal cortex does not seem to differentiate between the different types of kinaesthetic illusions, and its exact role in body perception remains unclear [43]. This is consistent with a variety of body-image disturbances that can be seen after lesions involving the inferior parietal cortex [44–47]. However, though the lesions probably included the inferior parietal cortex, they are typically very large, thus also including the superior parietal cortex.

Our findings also imply a functional–anatomical dissociation between the central representations of limb movement and perceived changes in the size and shape of body parts. Perception of passive limb movement engage frontal motor areas and parietal areas that are different [19,21,26,43,48] from those associated with changes in waist size (see also Figure S4). The reason for this dissociation probably relates to how the information is derived. Although limb movement can be represented by the analysis of afferent somatic input from a single limb in primary sensorimotor representations, the derivation of information about the relative size of body parts probably requires the integration of information from different body segments in higher-order areas.

The parietal activity can be associated with the illusory sensation that the size of the waist is changing, rather than with a complex kinaesthetic illusion involving two body parts more generally. Two points support this. First, we observed a significant relationship between the reported degree of illusory waist shrinking and the level of parietal activity (see the linear regression analysis and Figure 3). Second, in a previous imaging study [21] we studied effects of tendon vibration of the one wrist while both hands were mutually in contact palm to palm. This elicited an illusion that both hands were bending but without changes in body size [21,49]. Although this illusion also critically depends on the integration of somatosensory signals from different body parts (two limbs) and changes in position and orientation of the hand, we did not observe any activations in the parietal areas reported in the present study (*p* > 0.001 uncorrected using a sensitive fixed-effect analysis). Thus, the parietal activity is probably related to the illusory feeling that the size of the waist is changing.

We do not say that this activity is specific to waist shrinking as opposed to expansion. We predict the same activity for waist expansion because the BOLD signal cannot distinguish between the directions of movement. Likewise it is an open question as to what extent the location of the parietal activation we detected would depend on the body part that underwent the size-changes. Because the somatotopical organization of the posterior parietal cortex is coarse with extensive overlaps of the somatic receptive fields of different body parts, we would only predict small changes in the location of the activation peaks within the same parietal area when people feel that other body parts are shrinking.

It is important to clarify that our results cannot be explained by passive transduction of vibration from the vibration site to the waist and the abdomen. We know from the psychophysical experiments that passive spread from hands to abdomen does not cause any body-image illusions (e.g., during the skin contact condition). Moreover the spread of vibration was too weak to activate the posterior parietal cortex, as evident from the lack of activity in this area in the contrast skin contact – rest. Further, when we examined the interaction term, we were protected from effects related to passive spread because it occurred in both tendon contact and skin contact. The amount of passive spread in these conditions should be similar given that the vibrator is only moved 3–4 cm on the hands. Finally, passive transduction of vibration can never explain the correlation we observed between subjective ratings of the waist-shrinking illusion and the parietal activity.

In summary, we have shown that higher-order somatosensory areas in the junction between the postcentral and intraparietal sulci (probably areas 2/5) are involved in perceived changes in the size and shape of the waist. We suggest that the underlying mechanism is that these areas compute the relative size and shape of body parts by integrating multiple somatic signals from different body parts. Our finding is important because it provides direct neurophysiological evidence that the parietal cortex is involved in the construction of the body image.

## Materials and Methods

### Prescanning Psychophysical Test

We tested 24 blindfolded potential healthy subjects on the “shrinking-waist illusion” [4] in a separate experiment before the brain scans. All subjects were right handed and had given their informed consent. The local ethical committee had approved the study. We tested the same four stimulation conditions we later used in the brain scan (tendon contact, skin contact, tendon free, and skin free) as described in the section below on scanning. Each condition lasted 30 s and was repeated three times in a pseudo-randomized order. Seven subjects reported that they did not reliably feel the shrinking illusion (during tendon contact), and they were not scanned because the aim of the present study was to identify the neural correlates of perceived body-size changes.

In the condition in which the hands were in contact, the subjects were requested to indicate the onset of the illusion that the waist was shrinking by making a verbal response (saying “now”). An experimenter timed this response using a stopwatch. After the 30-s period of vibration of both wrists, the subjects were first asked “What did you feel?” and we noted the response. Then we asked them to select one picture out of six different body configurations that best corresponded to their experiences during the stimulation. The relevant picture showed the illusion of waist shrinking; the control pictures showed (1) waist enlargement, (2) hands moving into the waist but no waist shrinking, (3) longer arms, (4) shrinking torso and head, and (5) no changes in body image or hand movement. By these questions we could confirm that the participants experienced the shrinking-waist illusion. The subjects that felt the illusion were also asked to rate the vividness and continuance of the illusion on an analogue scale from 0 to 9. The vividness was defined as how realistic the illusion was when it was experienced (9 being “absolutely realistic”). The continuance score reflected the persistence of the illusion (equivalent to the percentage of time that the illusion was experienced). We also quantified the degree of perceived change in waist size. Directly after the 30-s vibration period, the subjects were asked to display the maximum perceived displacement of the wrists by holding the hands just above the body and flexing the wrists. We measured the angle of illusory wrist displacement and the distance between hands. These measurements reflect the degree of waist shrinkage because the subjects felt as if the waist was shrinking as much as the hands were bending inwards.

During these tests, we simultaneously recorded EMGs during the tendon contactand tendon free conditions (the ECR and the FCR of both forearms). We used a pair of 8-mm diameter Ag/AgCl electrodes (NT-211U; Nihon kohden, Tokyo, Japan) and an amplifier (AB-610J; Nihon kohden) for the digital registration and analysis of the muscle signals (PowerLab/16SP; ADInstruments, Sydney, Australia). We then calculated the integrated EMG (iEMG) to quantify the muscle activity during the different conditions.

### Brain Scanning: Experimental Design

On the basis of the results from the initial psychophysical testing described above, 17 subjects (four female, age 20 to 35 y; mean = 24 ± 3.2 y) were selected to take part in the fMRI experiment. Whilst the scanning was being performed, the blindfolded subjects rested comfortably in a supine position on the bed in the MRI scanner. We used two non-magnetic vibrators that were driven by constant air pressure provided by two air-compressors (Umihira Ltd, ILLUSOR, Kyoto, Japan). The frequency was approximately 110 Hz (amplitude: ± 3.5 mm) and the skin surface vibrated was approximately 1 cm^2^. Two experimenters in the scanner room manually operated the vibrators by applying them to the skin with a light pressure. To provide the two experimenters with synchronized instructions about the conditions and the onset and offset of the vibration, computer-generated visual cues were projected onto the white surface of the scanner (the blindfolded participants could not see this visual information). The participants were instructed to relax during the scans and not to make any movements.

There were four experimental conditions and two resting baselines. In tendon contact, both of the subject's hands were attached to the lateral sides of the waist and legs, palm to body. We vibrated the skin surface over the left and right tendons of ECR muscles (the muscle that extends the wrist). This stimulation causes a kinaesthetic illusion that both wrists are passively flexing and that the waist and upper parts of the legs are shrinking (see Figure 1A). In skin contact,the subjects had their hands in contact with the body (exactly as in tendon contact) but we vibrated the skin over the left and right processes styloideus ulnae, i.e., the skin beside the tendon. In this condition the subjects felt no illusion [21]. In the tendon freeand skin freeconditions, the hands did not touch the body but were positioned in a semi-pronated position so that the palms were towards the lateral sides of the body but not touching it (10-cm distance). In tendon free, we vibrated the tendon of the right and left ECR muscles, which caused the subjects to experience an illusory flexion of both wrists (but no change in body size; see Figure 1B). In skin free, we vibrated the skin beside the tendon over the processes styloideus ulnae, and the participants felt no illusion. Finally, in rest contactand rest free,we did not apply any vibratory stimuli and the subjects had their hands either in contact with the body (rest contact) or not in contact with the body (rest free). To reveal the activity that reflected the shrinking-body illusion, we examined the interaction between site of vibration and hand position using a 2 × 2 factorial design ([tendon contact – skin contact] – [tendon free – skin free]; see also Figure 1). The rationale of this design is that the interaction term reveals activity that reflects the shrinking body illusion and that cannot be attributed to the effects of vibrating the muscle tendon and hand position, i.e., to the sum of the main effects.

A complete experiment consisted of six experimental runs, each lasting 5 min and 36 s. In three runs, the hands lay freely beside the body without touching it and the arms were supported. In these runs we tested the three conditions tendon free, skin free, and rest free. In the three other experimental runs, the palms of the hands were in direct contact with the lateral sides of the body. A strap was used to attach the hands to the body, allowing the subjects to completely relax their arms. In these runs we collected data for the conditions tendon contact, skin contact, and rest contact. To eliminate time effects, the two types (free or contact) of experimental runs were performed in an alternating order that was counterbalanced across subjects.

Each condition lasted for 30 s. The vibration conditions were repeated three times in each run, and the rest condition was performed five times. During the runs we always had rest conditions before and after each vibration condition, and we alternated between tendon and skin vibration.

### Acquisition and Analysis of Functional Imaging Data

The functional imaging was conducted by using a Siemens Allegra 3.0T scanner (Erlangen, Germany) to acquire gradient echo T2*-weighted echo-planar images with BOLD contrast as an index of local increases in synaptic activity [50]. The image parameters used were: matrix size = 64 by 64, voxel size = 3 mm by 3 mm, echo time (TE) = 40 ms, and repetition time (TR) = 3,000 ms. A functional image volume comprised 48 slices of 3-mm thickness which ensured that the whole brain was within the field of view. For each of the six experimental runs (see above), we collected 112 image volumes, with one volume being collected every 3 s. A high-resolution T1-weighted structural image was also collected. The fMRI data was analysed using the Statistical Parametric Mapping Software [51] (SPM2, http://www.fil.ion.ucl.ac.uk/spm; Wellcome Department of Imaging Neuroscience, London, United Kingdom). The images were realigned to correct for head movements, co-registered with each subject's anatomical MRI, and transformed to the standard anatomical format. Thus, all coordinates refer to the standard space of the Montreal Neurological Institute (MNI) The functional images were spatially smoothed with a 10-mm full width at half maximum (FWHM) isotropic Gaussian kernel, and smoothed in time by a 4-s FWHM Gaussian kernel.

For each individual subject, we fitted a linear regression model (general linear model) to the data. Each condition was modelled with a boxcar function delayed by 4 s and convoluted with the standard SPM2 hemodynamic response function. Because we knew from the psychophysical test before the scans that the illusion of body shrinking started after 3.3 ± 2.0 s (see Results), we omitted the first 4 s of all conditions by defining these periods as conditions of no interest in the model. We defined linear contrasts in the general linear model to test our hypothesis. The result from this analysis was the estimated BOLD signals for this contrast from each of the 17 subjects (contrast images). To accommodate inter-subject variability, the contrast images from all subjects were entered into a random-effect group analysis (second-level analysis). One-sample *t*-tests were used (16 *df*). We used the threshold of *p* < 0.001 uncorrected in the whole brain. Because we had a priori anatomical hypothesis that the somatosensory section of the parietal cortex would be active, we also used a small volume correction in this region. We defined regions of interest using spheres of 20-mm radius around the most significant peaks of activity observed in the bilateral parietal cortex in the main-effect contrast of all four vibration conditions versus rest (*x* = −51, *y* = −42, *z* = 51, and *x* = 51, *y* = −42, *z* = 48). This main-effect contrast identifies somatosensory areas and can be used to define regions of interest because it is orthogonal to the interaction effect, i.e., statistically independent. The interaction effect in the left parietal cortex corresponded to *p* < 0.05 corrected. We do not report areas that did not show an increase in activity relative to rest (*p* < 0.01 uncorrected). In these cases the interaction effect was caused by a deactivation in the control stimulation conditions rather than an increase related to the shrinking-waist illusion.

Finally, to investigate the relationship between the strength of the illusion and the neural activity, we used linear regression analyses. Because we wanted to corroborate the results from the interaction analysis that had revealed activation in the parietal cortex, we restricted this analysis to the left and right parietal cortices. For each subject, we related the activity obtained during the shrinking-body illusion (interaction term) to the mean illusory displacement of the wrist in the tendon contact condition as measured in the tests conducted outside the scanner. This approach is valid because we knew from pilot experiments and our previous experiments that kinaesthetic illusions are consistent across test sessions within the same subject and that there are substantial differences in illusion strength between subjects [19]. First, we searched for active areas within the parietal lobes using the SPM2 regression model. This allowed us to identify the parietal region that showed the most significant relationship between illusion strength and fMRI activity. Second, we examined the relationship in exactly those two coordinates that corresponded to the peaks of the activations in the interaction analysis (see above).

The anatomical localization of the activations was related to the major sulci and gyri [52], distinguishable on a mean MRI generated from the standardized anatomical MRIs from the 17 subjects.

## Supporting Information

Figure S1 Quantification of the Illusions of Waist Shrinking and Waist Expansion The perceived changes in wrist angle when the hands were in contact palm to waist and the wrist flexor or extensor muscles were vibrated are shown. The illusory changes in the wrist angle (top row), in the distance between the hands (middle row), and the vividness of the illusion (lower row) are shown in the graphs. Error bars denote standard errors of means.
(2.7 MB TIF).

Figure S2 Quantification the Illusions of Head Shrinking and Head Expansion The perceived changes in wrist angle when the subjects put their right or left hand on top of their head and we vibrated the tendon of either the wrist flexor or extensor muscles are shown. The illusory changes in the wrist angle (top row), in the hand position (middle row), and vividness of the illusions (lower row) are shown. Error bars denote standard errors of means.
(851 KB TIF).

Figure S3 Analysis of EMG Data We compared the iEMG from the right and left ECR and the FCR muscles during the two experimental conditions in which we vibrated the muscle tendons (tendon contact; tendon free). Error bars indicate SD. The iEMGs were calculated for a vibration period of 30 s for each subject (*n* = 17 subjects who participated in fMRI study). There was no difference in muscle activity between the two conditions (paired *t-*tests; *p* > 0.05).
(2.8 MB TIF).

Figure S4 Activation Maps Associated with Feeling Illusory Hand Flexion and Waist Shrinking or Just Illusory Flexion of the Hands without Waist Shrinking Activation maps associated with feeling illusory hand flexion and waist shrinking are shown in the right column (tendon attached – skin attached); activation maps associated with the illusory flexion of the hands without waist shrinking are shown in the left column (tendon free – skin free). Note that the left intraparietal cortex (yellow circle) was activated only when subjects felt waist shrinking (top left) and not when they only felt that their wrists were bending (top right; compare with Figure 2). Further, activity associated with illusory wrist movements is seen bilaterally in the premotor cortex (top row), primary motor cortex (top row), and inferior parietal cortex (lower row).
(7.1 MB TIF).

Protocol S1 Psychophysical Experiment (31 KB DOC).

## Abbreviations

BOLD: blood oxygenation level-dependent
ECR: extensor carpi radialis
EMG: electromyogram
FCR: flexor carpi radialis
fMRI: functional magnetic resonance imaging
iEMG: integrated electromyogram
SD: standard deviation
