# Supplementary material for: Neural Substrate of Body Size: Illusory Feeling of Shrinking of the Waist
Source: PLoS Biol. 2005 Nov 29;3(12):e412. doi: 10.1371/journal.pbio.0030412 (PMC1287503; doi:10.1371/journal.pbio.0030412)
Supplement: Supplementary file 1 [file pbio.0030412.sd001.doc]

**Supporting Information**

*Psychophysical experiment*

To further examine and quantify the shrinking body illusion for two body parts we conducted a psychophysical experiment in 10 healthy subjects (8 right handed and 2 left handed). We wanted to access how the perceived size changes of a body part were related to the direction of illusory movement of the hand. The subjects lay on a bed in a supine position with their eyes closed. We used a vibrator which was set at 70 Hz (amplitude: ± 3.5mm).

In the first experiment we examined the illusion on the waist. As in the fMRI experiment the subject’s both hands were attached to the lateral sides of the waist and legs, palm-to-body. We vibrated the skin surface over the left and right tendons of the wrist extensor muscles (extensor carpi radialis) or the wrist flexor muscles (flexor carpi radialis). Each stimulation lasted 20 seconds and was repeated 3 times. After each stimulation period the participants were asked to verbally describe what they felt. Further, they were asked to display the maximum perceived displacement of the wrists by holding the hands just above the body and flexing the wrists. We measured the angle of illusory wrist displacement and measured the distance between the hands at two points (tip of the index fingers and base of the thumb). The subjects were also asked to rate the vividness and continuance of the illusion on a scale from 0 to 9. The vividness was defined as how life-like and realistic the illusion was when it was experienced (9 being ‘absolutely realistic’ and 0 being no illusion at all).

In the second experiment we tested the illusion involving the head. All procedures were the same as in the first experiment with the exception that this time the subjects had either the right or the left hand attached to the top of the head. When the subjects were asked to replicate the illusory movement of the hand in contact with the head, they were asked to hold their hand in front of their head. We measured the change in position of the tip of the index finger. This experiment was repeated both for the right and for the left hand.

In all cases the subjects felt that the only touched body part (head or waist) changed size and shape. No changes of other body parts or the whole body were reported. These size/shape changes always followed the direction of the illusory movement of the hand. When the hands were flexing (bending inwards) the subjects felt that the waist was shrinking, and when the hands were extending they felt the waist was expanding (see Figure S1). The same effect was observed for the head (Figure S2). The illusion only involved the body part that was in direct contact with the palm of the hands: when the hands were in contact with the waist the size/shape illusion was restricted to the waist region and when the hand was in contact with the head the illusion involves the head. Both the shrinking and the expansion illusions of the head and wrists were vivid (Figures S1 and S2). These observations confirm the general findings by Lackner et al. (1988) and provide new quantitative data about the illusion. The present findings show that the perceptual effects during the illusion are determined by the pattern of stimulation and the posture of the hands. Thus, the illusions are restricted to the body parts in contact with the hand(s). This suggests the mechanism of the illusion is the integration of somatic information from different limbs and the brain’s interpretation of the conflicting sensory inputs.
